# Editorial: Brain extracellular matrix: Involvement in adult neural functions and disease volume II

**DOI:** 10.3389/fnint.2022.1009456

**Published:** 2022-08-15

**Authors:** Harry Pantazopoulos, Sabina Berretta

**Affiliations:** ^1^Department of Psychiatry, University of Mississippi Medical Center, Jackson, MS, United States; ^2^Graduate Program in Neuroscience, University of Mississippi Medical Center, Jackson, MS, United States; ^3^Translational Neuroscience Laboratory, Mclean Hospital, Belmont, MA, United States; ^4^Department of Psychiatry, Harvard Medical School, Boston, MA, United States; ^5^Program in Neuroscience, Harvard Medical School, Boston, MA, United States

**Keywords:** extracellular matrix, substance use disorder (SUD), Alzheimer's disease (AD), spinal cord injury, sleep, synaptic plasticity

In recent years, the brain extracellular matrix (ECM) has come to the forefront of the neuroscience field. Its role as a key player in developmental and adult brain functions as well as in brain disease is being increasingly recognized. The ECM wide-ranging functions span an organism's lifetime, from early prenatal development to adulthood and aging (Ishii and Maeda, 2008; Cabungcal et al., 2013; Senkov et al., 2014; Suttkus et al., 2014; Maeda, 2015; Pomin, 2015; Karus et al., 2016; Carulli and Verhaagen, 2021; Fawcett et al., 2022). Growing interest in the brain ECM is leading to a greater understanding of the mechanisms underlying the ECM's role in synaptic plasticity, learning and memory (Senkov et al., 2014; Tsilibary et al., 2014; Beroun et al., 2019; Carulli and Verhaagen, 2021; Fawcett et al., 2022), regulation of neuronal activity (Wingert and Sorg, 2021), neurodevelopmental processes such as neuronal migration and axon guidance (Pizzorusso et al., 2002; Ishii and Maeda, 2008; Mauney et al., 2013; Maeda, 2015; Karus et al., 2016), neuroprotection (Morawski et al., 2004; Cabungcal et al., 2013; Suttkus et al., 2014), and hypothalamic regulation of metabolism (Dingess et al., 2018; Mirzadeh et al., 2019). Mounting evidence implicates the ECM in a growing number of brain disorders including schizophrenia, bipolar disorder, autism (Eastwood and Harrison, 2006; Ma et al., 2009; Weiss et al., 2009; Abdallah et al., 2012; Poelmans et al., 2013; Pantazopoulos and Berretta, 2016; Velmeshev et al., 2019; Brandenburg and Blatt, 2022), substance use disorders (Slaker et al., 2016; Garcia-Keller et al., 2019; Seney et al., 2021), depression (Riga et al., 2017; Alaiyed et al., 2020; Koskinen et al., 2020; Blanco and Conant, 2021), Alzheimer's disease (Scholefield et al., 2003; Schworer et al., 2013; Morawski et al., 2014; Yang et al., 2017), and stroke (Hobohm et al., 2005; Hartig et al., 2017). This collection of original and review articles in Volume II of this Special Issue, includes examples of novel ECM structures and the ECM's role in plasticity, axonal regeneration, neural injury, neuroinflammation as well as a broad range of brain disorders, providing a window into a growing but still largely unexplored field of investigations.

For several decades, Wisteria floribunda agglutinin (WFA) lectin labeling, first introduced in the 1990's by Hartig et al. (1992) has been the primary method for studying the ECM, and more specifically a distinct population of perineuronal nets (PNNs). Indeed, many important studies using WFA labeling have provided information on the functional of role of these ECM structured forms, their association with distinct neuronal populations and involvement in brain disorders. In this special issue, Hartig et al., provide an extensive perspective on methodological aspects related to WFA lectin for PNN labeling, based on 30 years of experience. Several methodological factors potentially impacting PNN detection with WFA are discussed, along with technical guidelines and a PNN labeling “toolbox”. Notably, applications for *in vivo* and *in vivo* labeling as well as electron microscopy are discussed. At the same time, the authors share important considerations related to experimental conditions that may affect WFA ECM labeling and introduce bias. The authors point to intriguing discrepancies between WFA labeling and its putative main target, i.e., aggrecan-associated chondroitin sulfate chains, discuss some of the factors potentially contributing to this discrepancy and emphasize the need for use of multiple PNN markers.

Much of the focus on brain ECM studies has been on PNNs, specialized structures first described by Camillo Golgi in 1898 (Golgi, 1989). PNNs ensheath distinct populations of neurons and regulate key neural functions such as synaptic plasticity, electrophysiological properties and access to neuroactive molecules (Kalb and Hockfield, 1988; Sugiyama et al., 2008; Gogolla et al., 2009). Emerging evidence shows that PNNs are only one of many structured ECM forms in the brain, including perisynaptic and perinodal ECM (Dours-Zimmermann et al., 2009; Bekku and Oohashi, 2010; Faissner et al., 2010; Frischknecht and Seidenbecher, 2012; Fawcett et al., 2019), chondroitin-6-sulfate clusters (CS6-clusters) and axonal coats (Hayashi et al., 2007; Bruckner et al., 2008; Okuda et al., 2014; Pantazopoulos et al., 2015). Evidence from the original manuscript by Pantazopoulos et al. shows that the chondroitin sulfate proteoglycans (CSPGs) NG2 and brevican form axonal coats, i.e., structured ECM sheaths, surrounding myelinated axons in the human thalamus. Detailed confocal and electron microscope analyses demonstrate intricate interweaving patterns of CSPGs with myelin sheaths and a preferential relationship with large axons. These findings, consistent with a role of brevican and NG2 in the regulation of axonal functions such as saltatory conductance and fasciculation, add to emerging evidence for a variety of ECM structures in the brain, likely to support specialized functions (see also Ray et al.).

The regulation of axonal functions, and specifically axonal growth, has been intensively studied in the context of axon injuries (Miyata and Kitagawa, 2015; Hussein et al., 2020). During neurodevelopment the ECM guides axons to their targets and regulates fasciculation (Snow et al., 2003; Kwok et al., 2012). In contrast, during adulthood, the ECM inhibits axonal regrowth following injuries (Miyata and Kitagawa, 2015; Hussein et al., 2020). Takiguchi et al. offer an important contribution to this field of investigations. Their elegant studies address an important question, i.e., whether growing axons following an injury reach and synapse with their targets in animals treated with ECM enzymatic degradation. Their results indeed demonstrate that, in experimental animals with complete spinal resection, enzymatic ECM degradation led to axonal growth through the injured site, formation of synapses between the newly grown axons and motor neurons, and improved motor functions.

ECM functions are in large part mediated by CSPGs and heparan sulfate proteoglycans (HSPGs). These large molecules are composed by a protein core to which a variable number of disaccharide chains are attached. The specificity of their biological effects is mediated by the sulfation patterns of the sugar chains, which determine their conformation, charge, and molecular affinities (Miyata et al., 2012; Smith et al., 2015). The elegant review contributed by Fawcett and Kwok focuses on the functional role of disaccharide sulfation on CSPGs and HSPGs. These latter have received less attention in the context of investigations on the brain ECM. Evidence compellingly reviewed by the authors highlights and compares CSPG and HSPGs functional roles in memory processing, neural injury, aging, axonal regeneration, and their modulation encoded by sulfation patterns.

The considerations above, particularly those emphasized by Hartig et al. and Fawcett and Kwok, resonate with those poignantly made by Scarlett et al. in the context of studies on the role of the ECM in brain disorders, and particularly Alzheimer's disease. As the authors eloquently point out, changes in the ECM sulfation “coding,” together with technical limitations surrounding traditional methods for PNN analyses, may reside at the root of important discrepancies in literature on the role of the ECM in neurodegenerative disorders. The paper puts forth the intriguing hypothesis that disease state-related chondroitin sulfation changes may impact PNN detection, and in turn interpretations of results showing altered PNN representation (Baig et al., 2005; Crapser et al., 2020). The distinction between altered PNN numbers and altered chondroitin sulfation patterns is significant, as each can affect CSPG functions, such as their ability to restrict or promote synaptic plasticity, in a different manner (Yang et al., 2021).

Inflammation has rapidly taken center stage as a key factor in a growing number of brain disorders, from autism to dementias, motor, and psychiatric disorders. The blood-brain barrier functions as a key interface regulating molecular transport between the brain and the peripheral circulatory system. In this special issue, Tabet et al. offer a comprehensive description of the relationship between the ECM and several components of the blood-brain barrier including the basement membrane and the glycocalyx. The authors discuss the role of the ECM in modulating blood-brain barrier permeability and its mediation of blood-brain barrier alterations during brain injury and inflammation.

Investigations on the ECM role in the pathogenesis of opioid use disorder (OUD) are rapidly gathering pace. Ray et al. offer a compelling overview of the ECM functional role within reward circuits and its involvement in OUD, including opioid seeking, craving and relapse. The authors consider the role of several forms of ECM, a recurring and important theme in this special issue. Particularly notable is the emphasis on sex-specificity, proposed to play a role both on the effects of opioid exposure on the ECM and on the contribution of ECM molecular signaling to opioid use disorder.

A novel and intriguing report by Liu et al. provides evidence for a role of the ECM in insomnia. The authors analyzed serum samples from patients with insomnia and demonstrated significant changes impacting several ECM factors, including matrix metalloproteinase 9 (MMP9) in persons suffering from insomnia. These findings bring forth a potential role for the ECM in sleep regulation. The intersection of ECM and immune factors found to be altered in insomnia offers new avenues for investigations on sleep deficits and potential therapeutic targets.

We believe that the articles highlighted in this special issue provide an exciting overview of the current state of research on the role of the ECM in the regulation of brain processes and involvement in disease states, and current directions regarding PNN labeling novel ECM structures. We hope that they will stimulate further studies the role of ECM in adult brain processes and psychiatric disorders. A deeper understanding of the role the ECM in these processes has the potential to allow us to leverage this system for preventative and therapeutic treatments.

## Author contributions

All authors listed have made a substantial, direct, and intellectual contribution to the work and approved it for publication.

## Conflict of interest

The authors declare that the research was conducted in the absence of any commercial or financial relationships that could be construed as a potential conflict of interest.

## Publisher's note

All claims expressed in this article are solely those of the authors and do not necessarily represent those of their affiliated organizations, or those of the publisher, the editors and the reviewers. Any product that may be evaluated in this article, or claim that may be made by its manufacturer, is not guaranteed or endorsed by the publisher.
